# Macrophage-derived macrophage migration inhibitory factor mediates renal injury in anti-glomerular basement membrane glomerulonephritis

**DOI:** 10.3389/fimmu.2024.1361343

**Published:** 2024-05-23

**Authors:** Hui Yang, Jinhong Li, Xiao-ru Huang, Richard Bucala, Anping Xu, Hui-Yao Lan

**Affiliations:** ^1^ Department of Nephrology, Sun Yat‐Sen Memorial Hospital, Sun Yat‐Sen University, Guangzhou, China; ^2^ Department of Medicine and Therapeutics, Li Ka Shing Institute of Health Sciences, Lui Che Woo Institute of Innovative Medicine, The Chinese University of Hong Kong, Hong Kong, Hong Kong SAR, China; ^3^ Department of Nephrology, The Seventh Affiliated Hospital of Sun Yat‐sen University, SunYat‐sen University, Shenzhen, China; ^4^ Departments of Nephrology and Pathology, Guangdong Provincial Hospital, Southern Medical University, Guangzhou, China; ^5^ Department of Internal Medicine, Yale University School of Medicine, New Haven, CT, United States

**Keywords:** macrophages, MIF, T cells, anti-GBM crescentic glomerulonephritis, inflammation

## Abstract

Macrophages are a rich source of macrophage migration inhibitory factor (MIF). It is well established that macrophages and MIF play a pathogenic role in anti-glomerular basement membrane crescentic glomerulonephritis (anti-GBM CGN). However, whether macrophages mediate anti-GBM CGN via MIF-dependent mechanism remains unexplored, which was investigated in this study by specifically deleting MIF from macrophages in MIF^f/f−lysM−cre^ mice. We found that compared to anti-GBM CGN induced in MIF^f/f^ control mice, conditional ablation of MIF in macrophages significantly suppressed anti-GBM CGN by inhibiting glomerular crescent formation and reducing serum creatinine and proteinuria while improving creatine clearance. Mechanistically, selective MIF depletion in macrophages largely inhibited renal macrophage and T cell recruitment, promoted the polarization of macrophage from M1 towards M2 via the CD74/NF-κB/p38MAPK-dependent mechanism. Unexpectedly, selective depletion of macrophage MIF also significantly promoted Treg while inhibiting Th1 and Th17 immune responses. In summary, MIF produced by macrophages plays a pathogenic role in anti-GBM CGN. Targeting macrophage-derived MIF may represent a novel and promising therapeutic approach for the treatment of immune-mediated kidney diseases.

## 1 Introduction

Anti-glomerular basement membrane crescentic glomerulonephritis (anti-GBM CGN) is an autoimmune glomerular disease that progresses rapidly. It is characterized by glomerular crescentic formation and the presence of autoantibodies that target specific epitopes on the a3 chain of type IV collagen (1–3). The development of anti-GBM CGN involves different cellular components, such as macrophages, lymphocytes, intrinsic renal cells, and a complex network of cytokines (4, 5). Despite extensive research, the precise mechanisms underlying this disease are still not fully understood.

Macrophages play a crucial role in the development of anti-GBM GN by infiltrating the affected kidneys and contributing to inflammation and fibrosis (6). Recent studies have shown that depletion of macrophages or inhibiting the production of cytokines by macrophages can alleviate kidney injury in anti-GBM CGN (7–9). The severity of the disease is associated with the infiltration and activation of macrophages, which can exhibit different phenotypes depending on the local environment. Pro-inflammatory M1 macrophages promote renal injury, whereas anti-inflammatory M2 macrophages offer protection against kidney diseases (6, 10–14).

Macrophage migration inhibitory factor (MIF) is a versatile proinflammatory cytokine that plays a crucial role in triggering the release of multiple downstream cytokines and facilitating the recruitment of leukocytes to inflammatory organs by binding to CD74 (15, 16). In the pathogenesis of murine autoimmune glomerulonephritis (GN), including anti-GBM CGN, MIF has been identified as a key player, influencing both the inflammatory and adaptive immune responses (17–21). Recent research demonstrated that mice lacking the MIF gene were protected from renal injury in a murine CGN model. Additionally, studies involving bone marrow reconstitution revealed that the absence of MIF from both bone marrow-derived and non-myeloid-derived sources improves experimental anti-GBM GN (22). However, further investigation is required to establish the specific contribution of macrophage-derived MIF in the context of anti-GBM CGN.

In order to investigate the potential role of macrophage-derived MIF in anti-GBM CGN and to uncover the underlying mechanisms, we utilized genetic techniques to create a conditional knockout of MIF specifically in macrophages. Through comprehensive evaluations, we determined the role of macrophage-derived MIF in the development of anti-GBM CGN. In addition, we also aimed to elucidate the underlying mechanisms of macrophage-derived MIF in the pathogenesis of anti-GBM CGN.

## 2 Results

### 2.1 Characterization of macrophage-specific MIF deficient mouse

**Figure 1:**
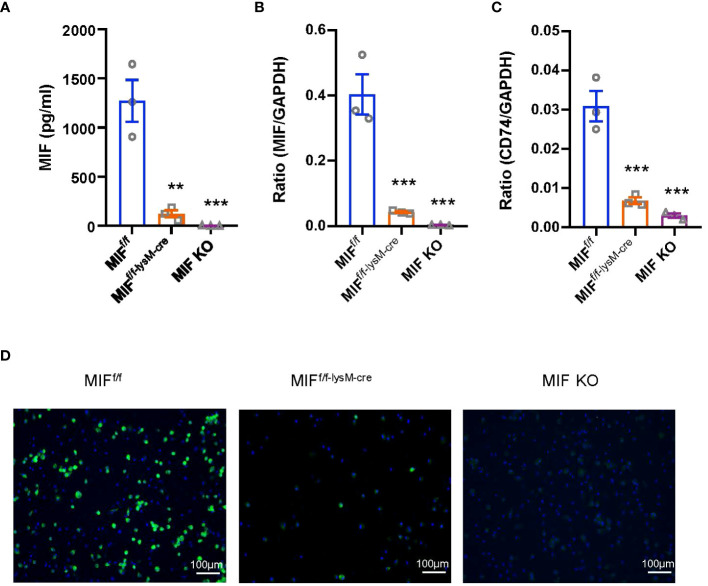
Characterization of MIF^f/f-lysM-cre^ mice. **(A)** Bone marrow-derived macrophages (BMDMs) were isolated from MIF^f/f^, MIF^f/f-lysM-cre^ and MIF KO mice. Enzymelinked immunosorbent assay (ELISA) show that MIF^f/f-lysM-cre^ and MIF KO mice inhibit MIF expression by BMDMs. **(B, C)** RNA was isolated from cells for quantitative reverse transcription-PCR (qRT-PCR) quantitation of MIF and CD74. **(D)** Immunofluorescence staining for MIF(green) with nuclear DAPI (blue) counterstain in macrophages stimulated with TNF-a (10ng/ml for 24hours). Each bar represents mean±SEM. Each dot represents one mouse. **P < 0.01, ***P < 0.001 compared with MIF^f/f^ mice; GAPDH, glyceraldehyde-3-phosphate dehydrogenase.

To evaluate the pathogenic role of macrophage-derived MIF, we employed a Cre-loxP strategy to generate mice with a specific deletion of MIF within their macrophages. To confirm the efficiency of MIF deletion, bone marrow was isolated from MIF^f/f-lysM-cre^ mice, control MIF^f/f^ mice, and MIF KO littermates. The isolated bone marrow cells were cultured and induced to differentiate into macrophages. As shown in **Figure 1A**, the deficiency of macrophage-derived MIF led to a notable reduction in MIF secretion by BMDM (**Figure 1A**). Additionally, MIF and CD74 mRNA expression were significantly lower in macrophages of MIF^f/f-lysM-cre^ mice compared with MIF^f/f^ mice under basal conditions (**Figures 1B, C**). Furthermore, TNF-a treatment for 24 hours significantly increased MIF protein expression in macrophages derived from MIF^f/f^ mice but not in macrophages from MIF^f/f-lysM-cre^ mice (**Figure 1D**). These results demonstrated the successful deletion of MIF from macrophages in MIF^f/f-lysM-cre^ mice.

### 2.2 Selective MIF depletion in macrophages ameliorates experimental anti-GBM GN

**Figure 2:**
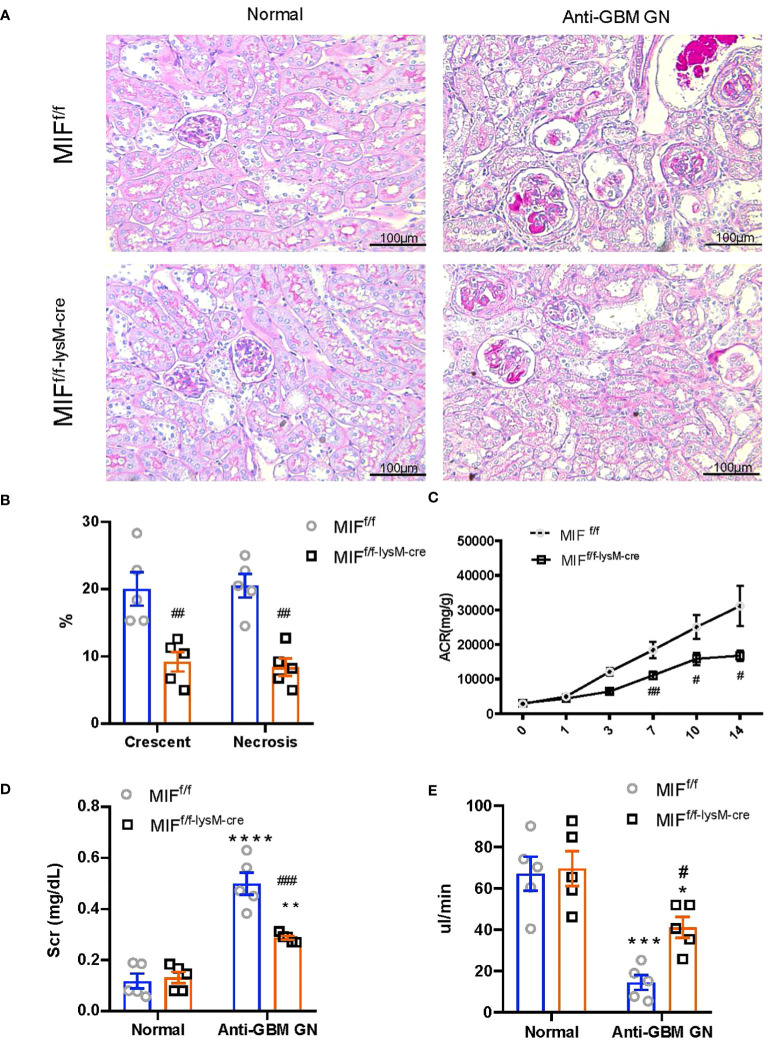
Selective MIF depletion in macrophages ameliorates experimental anti-GBM GN. **(A)** Representative images in PAS sections (magnification X200). **(B)** Semi-quantitative analysis of histology. **(C)** Urine albumin creatinine ration (ACR) over the disease course. **(D)** plasma creatinine. **(E)** Creatinine clearance. Each bar represents mean±SEM. Each dot represents one mouse. *p <0.05, **p <0.01, ***p< 0.001, ****p< 0.0001 versus corresponding control; ^#^p<0.05, ^##^p < 0.01, ^###^p< 0.001 versus corresponding MIF^f/f^.

To examine the involvement of macrophage-derived MIF in experimental anti-GBM CGN, we conducted experiments using both MIF^f/f^ and MIF^f/f-lysM-cre^ mice. The mice were induced to develop anti-GBM CGN, and the renal injuries were evaluated. Notably, the MIF^f/f-lysM-cre^ mice showed a significant inhibition in renal injuries such as segmental glomerular capillary necrosis and crescent formation compared to the anti-GBM GN MIF^f/f^ mice (**Figures 2A, B**). Renal dysfunction such as the urine albumin/creatinine ratio (**Figure 2C**), serum creatinine levels (**Figure 2D**), and creatinine clearance (**Figure 2E**) were also significantly improved in MIF^f/f-lysM-cre^ mice compared to the anti-GBM CGN MIF^f/f^ mice. These findings indicate that macrophage-derived MIF plays a pathogenic role in anti-GBM CGN.

### 2.3 Deletion of macrophage MIF inhibits macrophage and T cell infiltration in a mouse model of anti-GBM CGN

**Figure 3:**
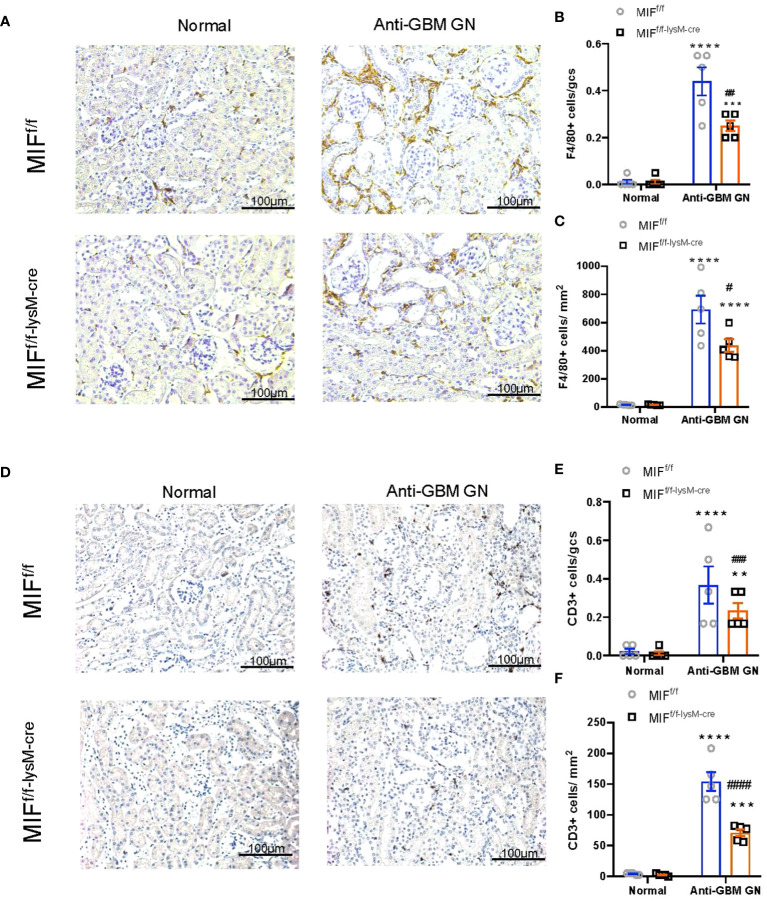
Selective MIF depletion in macrophages reduces macrophage and T cell recruitment in experimental anti-GBM GN. **(A)** Immunohistochemical staining for F4/80-positive macrophages in the kidney with anti-GBM crescentic GN on Day 14 after disease induction. **(B, C)** Summary data for macrophages in glomerulus and tubulointerstitium. **(D)** Immunohistochemical staining for CD3-positive T cells in the kidney with anti-GBM crescentic GN on Day 14 after disease induction. **(E, F)** Summary data for T cells in glomerulus and tubulointerstitium. Original magnification 200X. Each bar represents mean±SEM. Each dot represents one mouse. **p <0.01, ***p< 0.001, ****p< 0.0001 versus corresponding control; ^#^p<0.05, ^##^p < 0.01, ^###^p< 0.001, ^####^p<0.0001 versus corresponding MIF^f/f^.

We next examined macrophage-derived MIF on cellular immune response during anti-GBM CGN. Immunohistochemistry detected that a massive F4/80+ macrophages infiltrating the anti-GBM CGN in MIF^f/f^ mice, which was largely inhibited in MIF^f/f-lysM-cre^ mice (**Figures 3A-C**). Moreover, the infiltration of glomerular and interstitial CD3+ T cells in MIF^f/f-lysM-cre^ GN mice was also significantly lower than in MIF^f/f^ GN mice (**Figures 3D-F**). These findings demonstrate that selective depletion of MIF in macrophages suppresses the infiltration of macrophages and T cells in the kidney during anti-GBM GN.

### 2.4 Deletion of macrophage MIF suppresses antigen-specific antibody production in a mouse model of anti-GBM CGN

**Figure 4:**
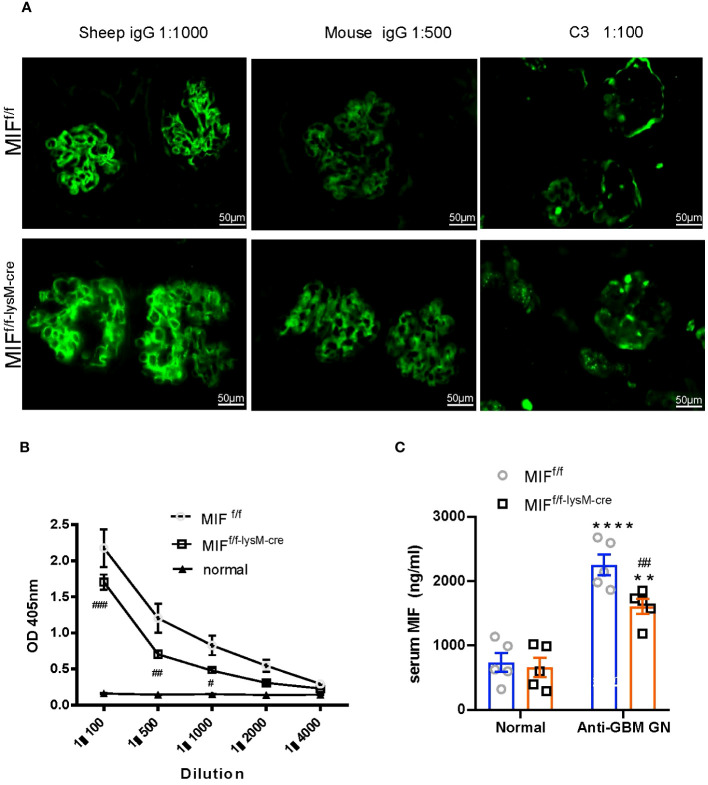
Selective MIF depletion in macrophages attenuates plasma levels of mouse anti-sheep IgG antibody and serum MIF production, which does not affect immune complex deposition in inflamed glomeruli. **(A)** Immunofluorescence staining for the deposition of sheep IgG, mouse IgG and mouse C3 in the kidney glomeruli with anti-GBM crescentic GN on Day 14 after disease induction. Plasma mouse anti-sheep IgG **(B)** and serum MIF **(C)** were determined using ELISA kits according to the manufacturer's protocol. Original magnification 200X. Each bar represents mean±SEM. Each dot represents one mouse. **p <0.01, ****p< 0.0001 versus corresponding control; ^#^p<0.05, ^##^p < 0.01, ^###^p< 0.001 versus corresponding MIF^f/f^.

Immunofluorescence was used to assess the glomerular deposition of sheep anti-mouse GBM antibody, mouse IgG, and complement component C3 in MIFf/f and MIFf/f-lysM-cre mice (**Figure 4A**). Interestingly, there was no significant difference in the glomerular deposition of these markers between the two groups, indicating that macrophage-specific MIF depletion did not affect immune complex deposition in inflamed glomeruli. However, as demonstrated in **Figure 4B**, the serum levels of mouse anti-sheep IgG antibodies were notably reduced in MIF^f/f-lysM-cre^ GN mice compared to MIF^f/f^ GN mice. This was associated with significant reduction in serum MIF levels in MIF^f/f-lysM-cre^ GN mice compared to MIF^f/f^ GN mice (**Figure 4C**). These results indicate that macrophage-specific MIF depletion reduced systemic MIF levels and selectively inhibited the antigen-specific antibody production without influencing the immune complex deposition in the inflamed glomeruli.

### 2.5 Deletion of macrophage MIF enhances macrophage polarization from M1 to M2 through in a mouse model of anti-GBM CGN

**Figure 5:**
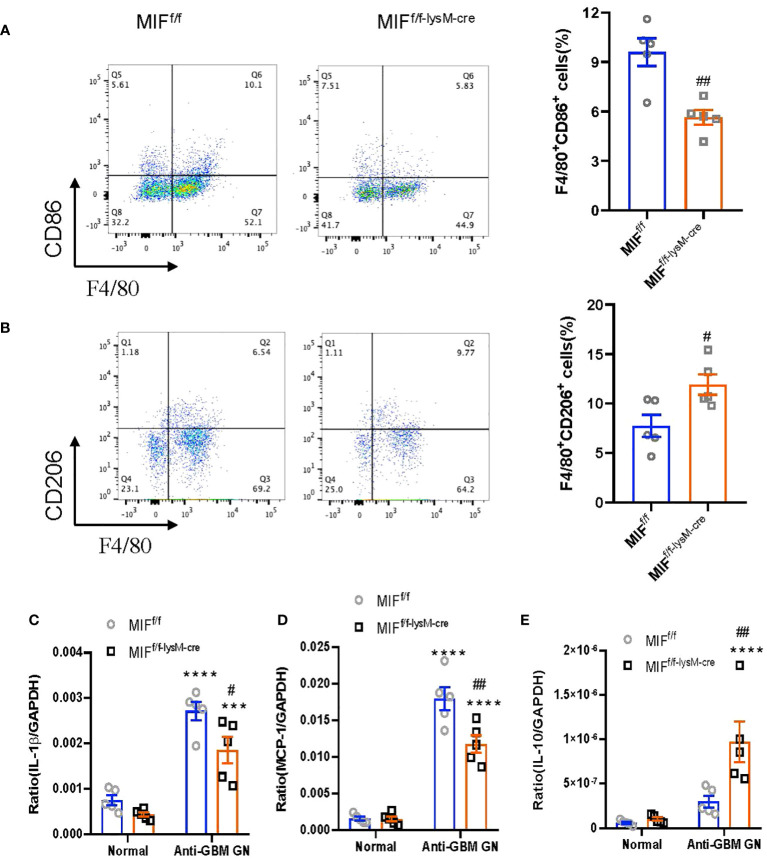
Selective MIF depletion in macrophages promotes macrophage polarization from M1 towards M2. **(A)** Representative flow cytometry plots and quantification of renal singlets analyzed for M1 (F4/80+CD86+). **(B)** Representative flow cytometry plots and quantification of renal singlets analyzed for M2 (F4/80+CD206+). Reverse transcription-PCR (RT-PCR) was performed on whole mouse kidney total RNA with anti-GBM crescentic GN on Day 14 after disease induction. **(C–E)** IL-1 β, monocyte chemoattractant protein-1 (MCP-1) and IL-10mRNA expression were normalized with GAPDH mRNA. Each bar represents mean±SEM. Each dot represents one mouse. ****p< 0.0001 versus corresponding control; ^#^p<0.05, ^##^p < 0.01 versus corresponding MIF^f/f^.

To investigate the impact of macrophage-derived MIF on macrophage polarization in the kidneys of mice with anti-GBM CGN, we utilized flow cytometry to determine the populations of M1 (F4/80+CD86+) and M2 (F4/80+CD206+) macrophages. Notably, MIF^f/f-lysM-cre^ GN mice exhibited a significantly decreased M1 macrophages while increasing the M2 macrophages compared to MIF^f/f^ GN mice (**Figures 5A, B**; **Supplementary Figure S1**). Furthermore, real-time PCR analysis revealed that selective MIF depletion in macrophages led to a significant inhibition of pro-inflammatory cytokines including MCP-1 and IL-1β while increasing the anti-inflammatory cytokine IL-10 in CGN mice (**Figures 5C–E**). These findings indicate that selective depletion of MIF in macrophages results in a shift in macrophage polarization from the pro-inflammatory M1 phenotype towards the anti-inflammatory M2 phenotype in experimental anti-GBM CGN. Moreover, this shift is associated with reduced pro-inflammatory cytokine expression and increased anti-inflammatory cytokine expression.

### 2.6 Deletion of macrophage MIF promotes Treg but inhibits Th1 and Th17 immune responses in a mouse model of anti-GBM CGN

**Figure 6:**
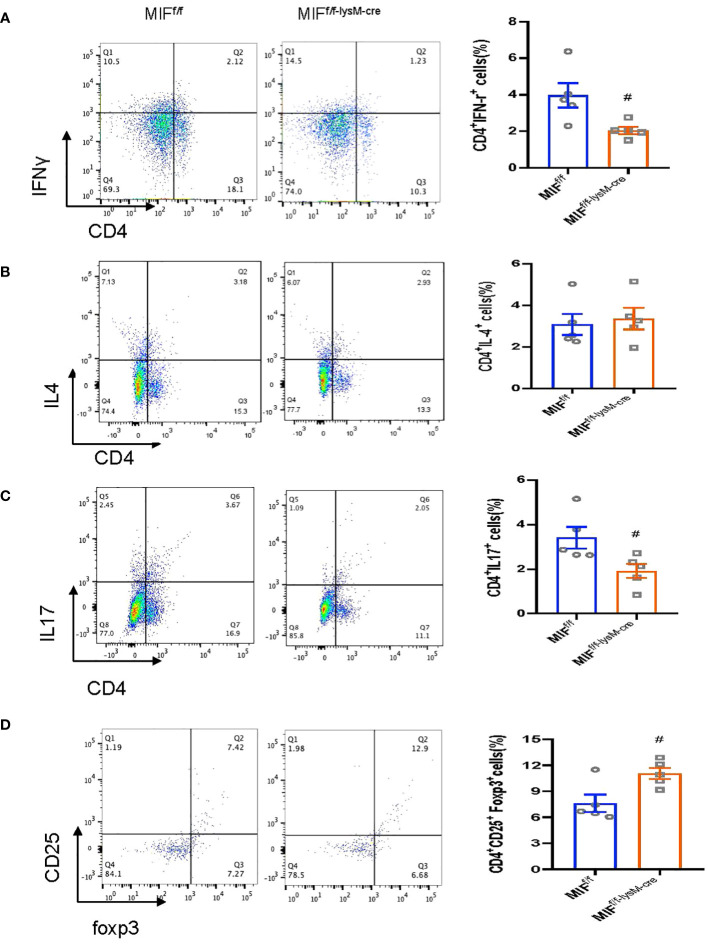
Selective MIF depletion in macrophages downregulates renal Th1/Th17 response and upregulates renal Treg response in experimental anti-GBM GN. Flow cytometry analysis of renal infiltrated CD4+IFNy+Th1 cells **(A)**, CD4+ IL-4+Th2 cells **(B)**, CD4+ IL-17+Th17 cells **(C)** and CD4+CD25+ Foxp3+Treg **(D)**. CD4+CD25+ Foxp3+Treg is Gated on CD4+ T cells. Each bar represents mean±SEM. Each dot represents one mouse. ^#^p<0.05 versus corresponding MIF^f/f^.

We next examined whether disrupted macrophage MIF influences T cell immunity as it is well-established that Th1 and Th17 are pathogenic whereas Treg is protective in anti-GBM CGN (23–26). In light of this, we investigated the impact of selective MIF depletion in macrophages on the immune differentiation of CD4+ T cells, specifically focusing on Th1 (CD4^+^IFNγ^+^), Th2 (CD4^+^IL-4^+^), Th17 (CD4^+^IL-17a^+^) and Treg (CD4^+^CD25^+^FoxP3^+^) subpopulations. Flow cytometry analysis revealed that selective MIF depletion in macrophages led to a significant reduction in Th1 (CD4^+^IFNγ^+^) and Th17 (CD4^+^IL-17a^+^) cells in the anti-GBM CGN kidney (**Figures 6A, C**; **Supplementary Figures S2A, C**). Conversely, there was an increase in Treg population (CD4^+^CD25^+^FoxP3^+^), however, the Th2 (CD4+IL-4+) immune response remained unaffected (**Figures 6D, B**; **Supplementary Figure S3**, **Supplementary Figure S2B**). These findings indicate that selective MIF depletion in macrophages enhances the Treg immune response while inhibiting the Th1 and Th17 immune responses in experimental anti-GBM CGN. This shift in immune cell differentiation may contribute to the amelioration of renal injury observed in this context.

### 2.7 *Deletion of macrophage MIF* inhibits Anti-GBM GN by inactivating M1 macrophages via CD74/NF-κB and p38 MAPK-dependent mechanisms *in vivo* and *in vitro*

**Figure 7:**
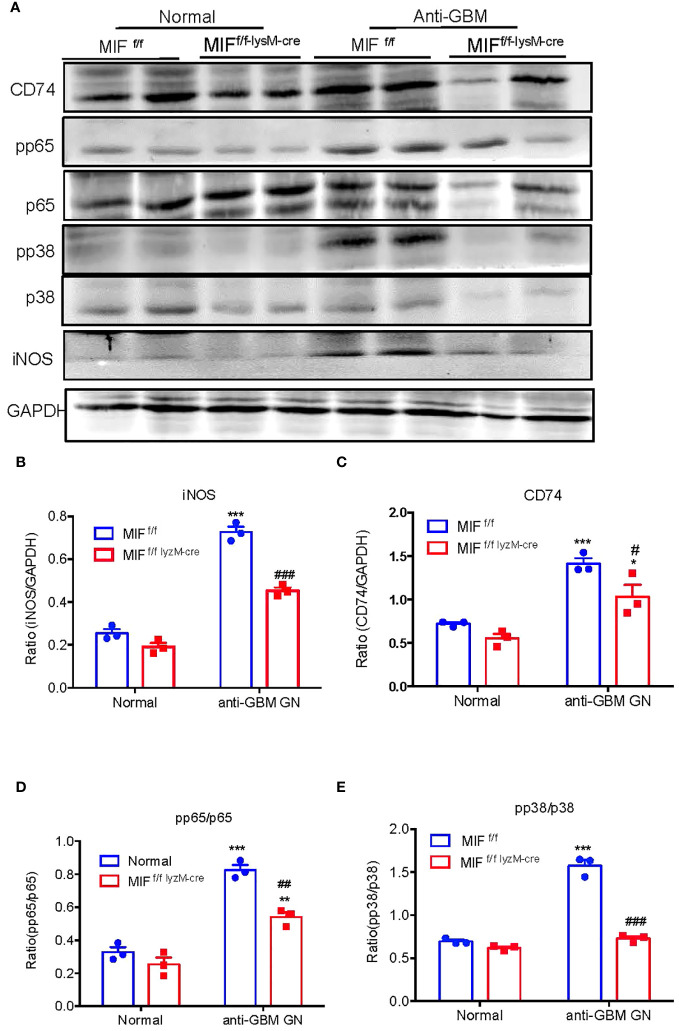
Selective MIF depletion in macrophages ameliorates experimental anti- GBM GN by inactivating NF-kB and p38/MAPK signaling and inhibiting M1 macrophages activation. **(A)** Western blots. **(B–E)** Statistics data of the protein expression (iNOS, CD74, pp65/p65 and pp38/p38). Each bar represents mean ± SEM. Each dot represents one mouse. *p <0.05, ***p<0.001 versus corresponding control; ^#^p<0.05, ^##^p < 0.01, ^###^p< 0.001 versus corresponding MIF^f/f^.

**Figure 8:**
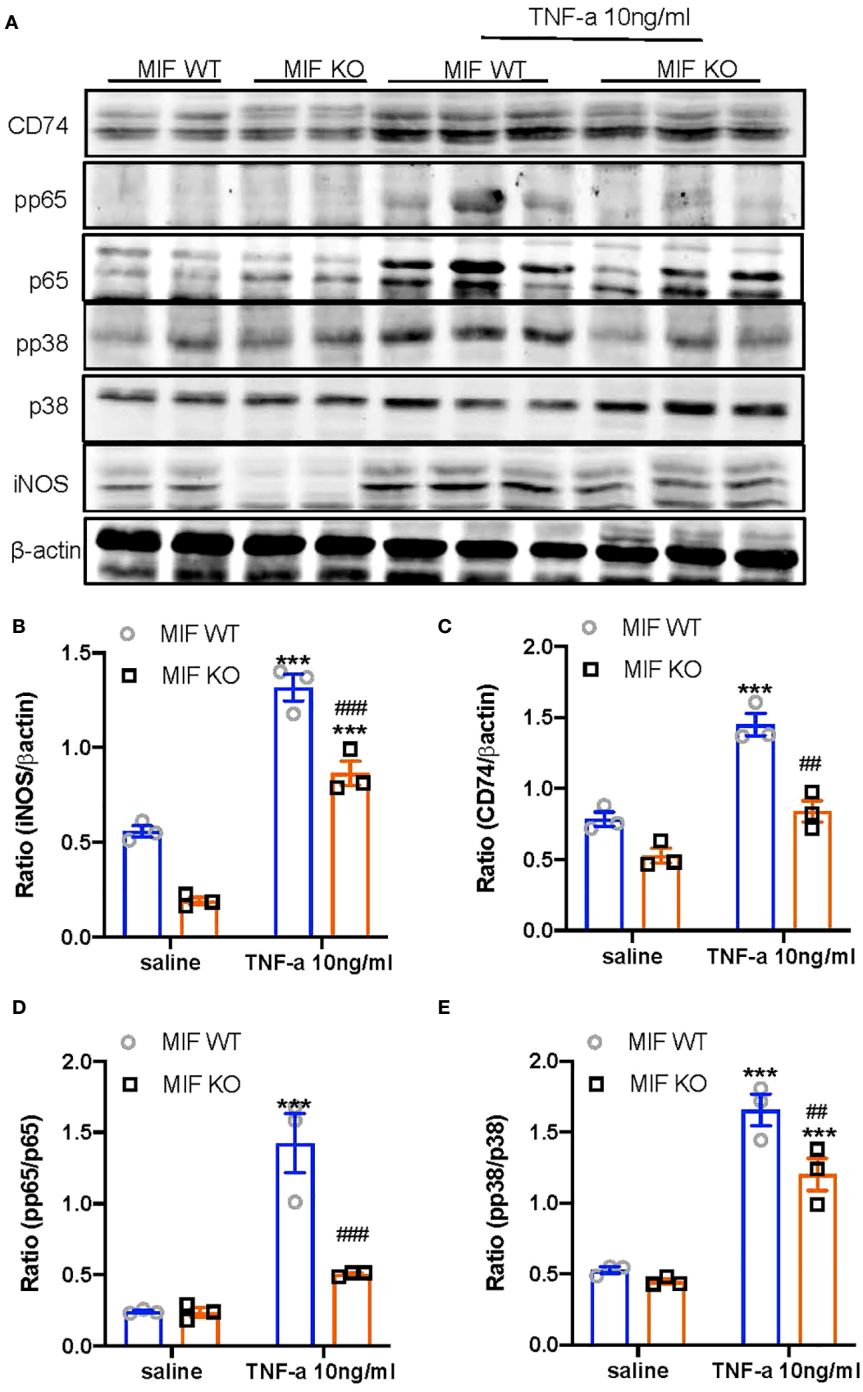
Bone marrow-derived macrophages (BMDM) lacking MIF show inhibition of MIF signaling and M1 macrophage activation in response to TNFa *in vitro*. **(A)** Western blots; **(B–E)** Statistics data of the protein expression (CD74, pp65/p65, pp38/p38, INOS). Results show that bone marrow-derived macrophages (BMDMs) isolated MIF KO mice inhibit TNF-α (10ng/ml)-induced upregulation of MIF signaling by downregulating CD74 expression and activation of NF-kB/p65 and p38/MAPK, there by inhibiting M1 macrophage activation by suppressing iNOS expression. Each bar represents mean±SEM. Each dot represents one mouse. ***P < 0.001 compared with MIF WT mice; ^##^P < 0.01, ^###^p< 0.001 compared with MIF WT mice treated with TNF-a 10ng/ml.

We next examined the mechanisms through which specific deletion of macrophage MIF inhibits anti-GBM GN induced in MIF ^f/f^ and MIF ^f/f-lysM-cre^ mice. Western blot analysis revealed that there was a marked upregulation of CD74 and activation of NF-κB/p65 and p38 MAPK signaling and expression of iNOS in the diseased kidney of MIF ^f/f^ mice (**Figure 7**). In contrast, selective MIF depletion from macrophages significantly inhibited the expression of CD74 and phosphorylation of NF-κB/p65 and p38 MAPK, as well as expression of iNOS in MIFf/f-lysM-cre GN mice (**Figure 7**). All of these findings indicated that MIF may promote anti-GBM GN by activating M1 macrophages through the CD74/NF-kB/p38 MAPK signaling. This was further demonstrated *in vitro* in cultured BMDM from MIF WT and MIF KO mice. We found that addition of TNF-α largely promoted iNOS-producing M1 macrophages in MIF WT BMDM by activating CD74/NF-κB/p38 MAPK signaling, which was blocked in BMDM lacking MIF (**Figure 8**). Thus, macrophage-derived MIF may mediate anti-GBM GN by promoting M1 macrophage activation via the CD74/NK-κB/p38 MAPK-dependent mechanism.

## 3 Discussion

MIF has been implicated in the pathogenesis of various diseases, including infectious diseases, inflammatory diseases, immune diseases such as rheumatoid arthritis, septic shock, and cardiovascular disease (20, 23–26). Previous studies have also demonstrated the importance of MIF in kidney-related conditions like acute kidney injury (AKI), chronic kidney disease (CKD), diabetic nephropathy, autosomal dominant polycystic kidney disease (ADPKD), and vasculitides (27–35).In glomerulonephritis, inhibition of MIF by neutralizing antibodies has shown renal protective in IgA nephritis and in rats with crescentic GN (36–38). Additionally, systemic MIF knockout (KO) can also suppress lupus nephritis and anti-GBM CGN (17, 19–22). However, it should be noted that systemic MIF KO models cannot distinguish the source of functional MIF since MIF is released by both macrophages and other intrinsic cells within the kidney. To address this, we generated mice with a macrophage-specific MIF KO and investigated the role and mechanisms of macrophage-derived MIF in a mouse model of anti-GBM GN. The results demonstrated that mice with macrophage-specific deletion of MIF were protected from the development of anti-GBM CGN. These findings highlight the crucial role of macrophage-derived MIF in the pathogenesis of anti-GBM CGN, shedding light on the specific contribution of macrophage-derived MIF in this disease context.

It is well-established that macrophages play a crucial role in the progressive renal injury associated with glomerular crescentic formation (6–8, 10, 11, 39). It is reported that MIF regulates macrophage activation via Toll-like receptor 4 (TLR4) (40). Thus, deletion of macrophage TLR4 inhibits anti-GBM cGN (39). It is also well defined that MIF is pathogenic in immunologically-mediated kidney disease as mice lacking MIF are protected against lupus mice and anti-GBM GN (17, 22). Similarly, systemic or bone marrow disruption of MIF also inhibits cardiac remodeling by suppressing myocardial leukocyte infiltration and the expression of inflammatory mediators (41). Findings from the present study added new information that macrophages may mediate anti-GBM CGN via MIF-dependent mechanism as selective depletion of macrophage MIF protected against anti-GBM CGN by inhibiting macrophage infiltration and promoting macrophage polarization from the pro-inflammatory M1 phenotype towards the anti-inflammatory M2 phenotype. These results suggest that macrophage-derived MIF may have a critical role in modulating macrophage activation and function in the pathogenesis of anti-GBM CGN.

Furthermore, an intriguing aspect of our findings is that the specific depletion of MIF in macrophages appears to confer kidney protection in crescentic GN by promoting renal Treg cells while suppressing Th1 and Th17 immune responses. The involvement of Th1 and Th17 immune responses in the pathogenesis of anti-GBM GN is well-established (42–44), whereas Treg cells are known to have a protective role (44, 45). In murine crescentic glomerulonephritis, Treg cells have been shown to regulate the Th1 immune response (45). Additionally, recent studies have highlighted the important role of cytokines and chemokines in the cross-regulation of Th1 and Th17 immune responses in experimental anti-GBM GN (46). Interestingly, MIF has been shown to enhance the acquisition of a Th17 cell-like phenotype in spondylarthritis (47). Based on these findings, it is reasonable to speculate that selective depletion of MIF in macrophages may protect against anti-GBM crescentic GN by suppressing Th1/Th17 while promoting Treg immune responses.

CD74 is identified as main receptor of MIF (48). The binding of MIF to CD74 triggers the activation of mitogen-activated protein kinase (MAPK) and NK-kB signaling (49, 50). It has been reported that M1 macrophage activation in anti-GBM GN is NF-κB-dependent (51) and M1-mediated NF-κB signaling can release cytokines including IL-1β, IL-6, TNF-α and granulocyte colony-stimulating factors (G-CSF) (52). The present study unraveled that deletion of macrophage MIF ameliorated anti-GBM GN by shifting the M1 macrophages to M2 macrophages via the CD74/NK-kB/p38 MAPK-dependent mechanism. Importantly, deletion of macrophage MIF inhibited the Th1 and Th17 immune responses, while increasing Treg. These findings also suggest that macrophage-derived MIF may play a regulatory role in T cell immunity during the development of anti-GBM GN, although the mechanisms remain largely unclear.

**Figure 9:**
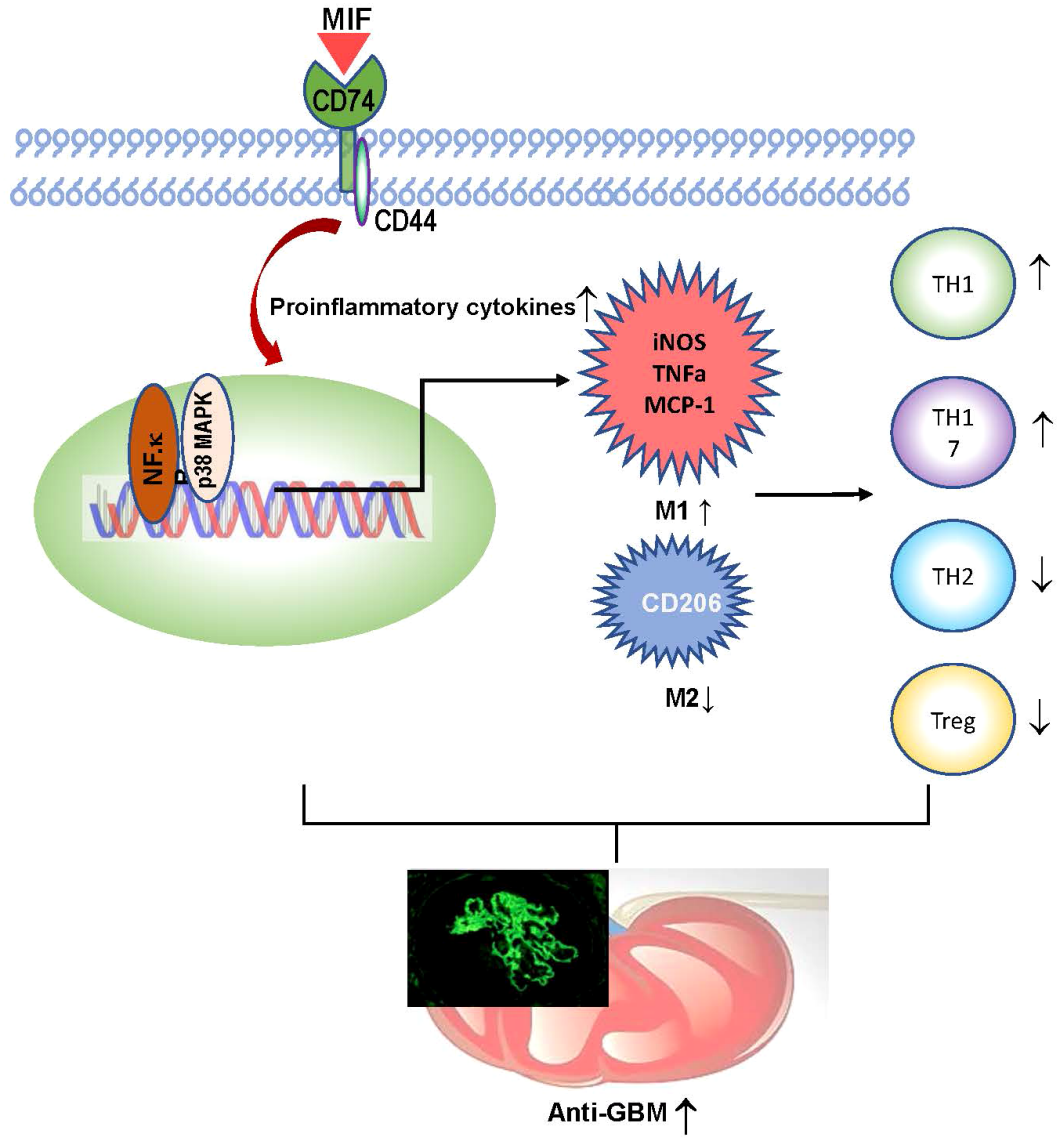
Mechanisms of MIF mediate experimental anti-GBM GN.

In summary, macrophage-derived MIF plays an important role in anti-GBM CGN. Mechanistically, as shown in **Figure 9**, macrophage-derived MIF may mediate anti-GBM CGN by promoting proinflammatory M1 macrophage infiltration and activation via the NK-κB and p38 MAPK pathways and promoting the Th1/Th17 immune responses while suppressing the Treg population. These findings are in line with our previous studies that T cell-mediated immunity plays a crucial role in anti-GBM disease, despite not necessarily impacting the glomerular deposition of immune complexes (44).

## 4 Materials and methods

### 4.1 Generation of macrophage-specific MIF deletion mice

To generate mice with myeloid-specific MIF deletion (MIF^f/f−lysM−cre^), we utilized C57BL/6 mice carrying MIF genes with homozygous loxP-flanked regions (MIF^f/f^), which were previously described (53). Lysozyme M promoter-driven cre (lysM-cre) mice were obtained from the Jackson Laboratory in the USA. The MIF^f/f^ mice were crossed with lysM-cre mice to obtain the desired genotype. The genotypes of the resulting littermates were confirmed using PCR with specific primers recommended by the Jackson Laboratory. All mice used in the study were maintained under specific pathogen-free conditions at a temperature of 25°C and a 12-hour light-dark cycle. They were housed in our animal facility and provided with standard food and water ad libitum.

### 4.2 Isolation and culture of bone marrow-derived macrophages

To culture bone marrow-derived macrophages (BMDMs), bone marrow cells were isolated from the tibias and femurs of MIF^f/f^, MIF^f/f−lysM−cre^, and MIF knockout (KO) mice. These cells were then cultured in Dulbecco modified Eagle medium (DMEM) supplemented with 50 ng/ml macrophage colony-stimulating factor (M-CSF) for 7 days, following a previously established protocol (54). After the 7-day culture period, BMDMs were stimulated with TNF-α (10 ng/ml) for 24 hours and proteins were collected for western blot analysis.

### 4.3 Real-time PCR analysis

Total RNA was extracted from either diseased kidney tissue or cultured cells using the RNeasy Isolation Kit (Qiagen, Valencia, CA) following the manufacturer’s protocol. RT-PCR was conducted following established methods (44, 55). The mRNA expression levels of the target genes were normalized to glyceraldehyde-3-phosphate dehydrogenase (GAPDH) as an internal control. The primer sequences for mouse MIF, CD74, MCP-1, IL-1β, IL-10, and GAPDH were previously reported (44, 55, 56).

### 4.4 Induction of anti-GBM glomerulonephritis in mice

The mouse model of anti-glomerular basement membrane crescentic glomerulonephritis (anti-GBM cGN) was established using male MIF^f/f^ and MIF^f/f−lysM−cre^ mice (8–12 weeks old), following a well-established protocol (44). Herein, the procedure involved the following steps: Firstly, groups of MIF^f/f^ and MIF^f/f−lysM−cre^ mice received a flank subcutaneous immunization with normal sheep IgG mixed with Freund’s complete adjuvant (Sigma Aldrich, St. Louis, Missouri, USA) five days prior to the initiation of the experiment. Anti-GBM cGN was induced by administering sheep anti-mouse GBM IgG via tail vein injection at a dose of 60 μg/g of body weight (referred to as day 0). On day 14, the mice were sacrificed using a lethal dose of ketamine and xylazine mixture. Age-matched normal male MIFf/f and MIFf/f−lysM−cre mice (n = 5 per group) were included as normal control counterparts. All animal experiments were conducted in compliance with the guidelines approved by the Animal Experimentation Ethics Committee at the Chinese University of Hong Kong.

### 4.5 Measurement of proteinuria and creatinine

Analysis of proteinuria: Urinary samples were collected at various time points, including before and after the induction of anti-GBM disease on days 0, 1, 3, 7, and 14. Proteinuria analysis was performed following the manufacturer’s protocols, as described previously (44). Urinary albumin excretion was quantified as total urinary albumin/creatinine ratio (expressed as micrograms per milligram). Measurement of urinary and serum creatinine levels was conducted using an enzymatic kit (Stanbio Laboratory, Boerne, USA).

### 4.6 Renal pathology and immunohistochemistry

Renal pathology assessment was conducted on methyl Carnoy’s fixed, paraffin-embedded tissue sections (4μm thick). The sections were stained with periodic acid Schiff (PAS) to visualize the renal structures. Glomerular crescents and necrosis were quantified by examining 50 glomeruli per diseased kidney section and calculating the percentage of affected glomeruli. Immunohistochemistry was performed on paraffin sections stained with monoclonal an anti-F4/80 antibody (Serotec, Oxford, UK) to detect macrophages, and a rabbit anti-mouse polyclonal CD3+ antibody (SP7) (Abcam, Cambridge, UK) to identify total T cells. The number of positive cells for CD3 and F4/80 was counted in 20 glomeruli and expressed as cells per glomerular cross-section (gcs). In the tubulointerstitium, positive cells were counted under high-intensity fields (400× magnification) using a 0.0625 mm^2^ graticule fitted in the microscope eyepiece. The cell count was then reported as cells per square millimeter (mm^2)^.

### 4.7 Immunofluorescence

To assess the presence of MIF-expressing macrophages in the kidney, immunofluorescence staining was performed. Acetone-fixed bone marrow-derived macrophages (BMDMs) were cultured with the MIF antibody (sc-20121; Santa Cruz) followed by incubation with a fluorescein isothiocyanate anti-rabbit secondary antibody, as previously described (54). To evaluate immune deposition in the glomeruli, direct immunofluorescence was conducted using FITC-conjugated polyclonal antibodies specific to sheep IgG, mouse IgG, and complement C3, following established protocols (38).

### 4.8 Enzyme-linked immunosorbent assay

Plasma levels of mouse anti-sheep IgG were quantified using a method previously described (56). The concentration of serum MIF was determined using ELISA kits (R&D Systems, Minneapolis, USA) according to the manufacturer’s instructions.

### 4.9 Flow cytometry analysis

Kidney single cells were prepared and subjected to flow cytometry analysis following a previously described protocol (44, 56). Single-cell suspensions were treated with IC Fixation Buffer and Permeabilization Buffer (eBioscience) to allow intracellular staining. The suspended kidney cells were then incubated with specific conjugated antibody cocktails in the dark for 30 minutes on ice. Negative controls included cells treated with irrelevant antibodies (isotype). Cells were also incubated with only one specific conjugated antibody. The antibodies used in this study were as follows: F4/80-Pacific blue (BioLegend, Catalog: 123124), CD86-APC (BioLegend, Catalog: 10512), CD206-Alexa 647 (Serotec, Catalog: MCA2235), CD4-FITC (eBioscience, Catalog: 11–0042-86), IFNγ-APC (eBioscience, Catalog: 17–7311-82), IL-4-PE (eBioscience, Catalog: 12–7041-82), IL-17a-PE (eBioscience, Catalog: 12–7177-81), CD25-PE (eBioscience, Catalog: 12–0251-83), and Foxp3-APC (eBioscience, Catalog: 12–0251-83). Flow cytometry analysis was performed using a FACS Calibar instrument and analyzed using the CellQuest Pro Analysis software (BD Biosciences, Franklin Lakes, New Jersey, USA).

### 4.10 Western blot analysis

Western blotting was performed as described previously (55, 57–59). Proteins from BMDMs and the kidney cortex were extracted with RIPA lysis buffer. After blocking nonspecific binding with 5% BSA, membranes were incubated overnight at 4 ° C with the primary antibodies against rabbit anti-iNOS (Abcam ab-15323), rabbit anti-iNOS (Abcam ab-178945), goat anti-CD74 (Santa Cruz, sc-5438), mouse anti-CD74 (Santa Cruz, sc-6267), goat anti-CD74 (Santa Cruz, sc-5438), rabbit anti-phosphorylated NF-kB p65 (Cell Signaling, #3031), rabbit anti-phosphorylated NF-kB p65 (Cell Signaling, #3033s), mouse anti-NF-kB p65 (Cell Signaling, #6965), rabbit anti-NF-kB p65 (Cell Signaling, # 8242S), rabbit anti-pp38 (Cell signaling, #9211), rabbit anti-pp38 (Cell signaling, # 4631L), rabbit anti-p38 (Cell signaling, #9212), rabbit anti-p38 (Cell signaling, # 8690S), mouse anti-ß-actin (Santa Cruz, sc-69879), mouse anti-GAPDH(proteintech,#60004–1-Ig).Then the membranes were incubated with IRDye800-conjugated secondary antibody (Rockland Immun- chemicals). Signals were scanned using the Odyssey IR imaging system (LI-COR Biosciences). Image J software (National Institutes of Health) was used for quantitative analysis of images.

### 4.11 Statistical analysis

Statistical analysis was conducted using Prism 9.0 GraphPad Software (GraphPad Software, La Jolla, California, USA). Data obtained from the study were presented as the mean ± standard error of the mean (SEM). Two-group comparisons were assessed using an independent sample t-test. Multiple group comparisons were performed using one-way analysis of variance (ANOVA) followed by Tukey’s *post hoc* tests. A p-value less than 0.05 was considered statistically significant in this experiment.

## 5 Conclusions

Our study demonstrates that selective MIF depletion in macrophages ameliorates experimental anti-GBM cGN by promoting macrophage polarization from M1 towards M2, enhancing Treg while inhibiting Th1 and Th17 immune responses via CD74/NF-κB/p38 MAPK signaling. Thus, targeting macrophage-derived MIF could be a novel therapy for anti-GBM cGN.

## Data availability statement

The raw data supporting the conclusions of this article will be made available by the authors, without undue reservation.

## Ethics statement

The animal study was approved by The Chinese University of Hong Kong. The study was conducted in accordance with the local legislation and institutional requirements.

## Author contributions

HY: Writing – original draft. JL: Writing – original draft. XH: Methodology, Writing – review & editing. RB: Methodology, Resources, Writing – review & editing. AX: Funding acquisition, Writing – review & editing. HL: Funding acquisition, Writing – review & editing.
