# Supplementary material for: Macrophage-derived macrophage migration inhibitory factor mediates renal injury in anti-glomerular basement membrane glomerulonephritis
Source: Front Immunol. 2024 May 23;15:1361343. doi: 10.3389/fimmu.2024.1361343 (PMC11153660; doi:10.3389/fimmu.2024.1361343)
Supplement: Supplementary file 1 [file Image_1.pdf]

Supplementary Figure 1

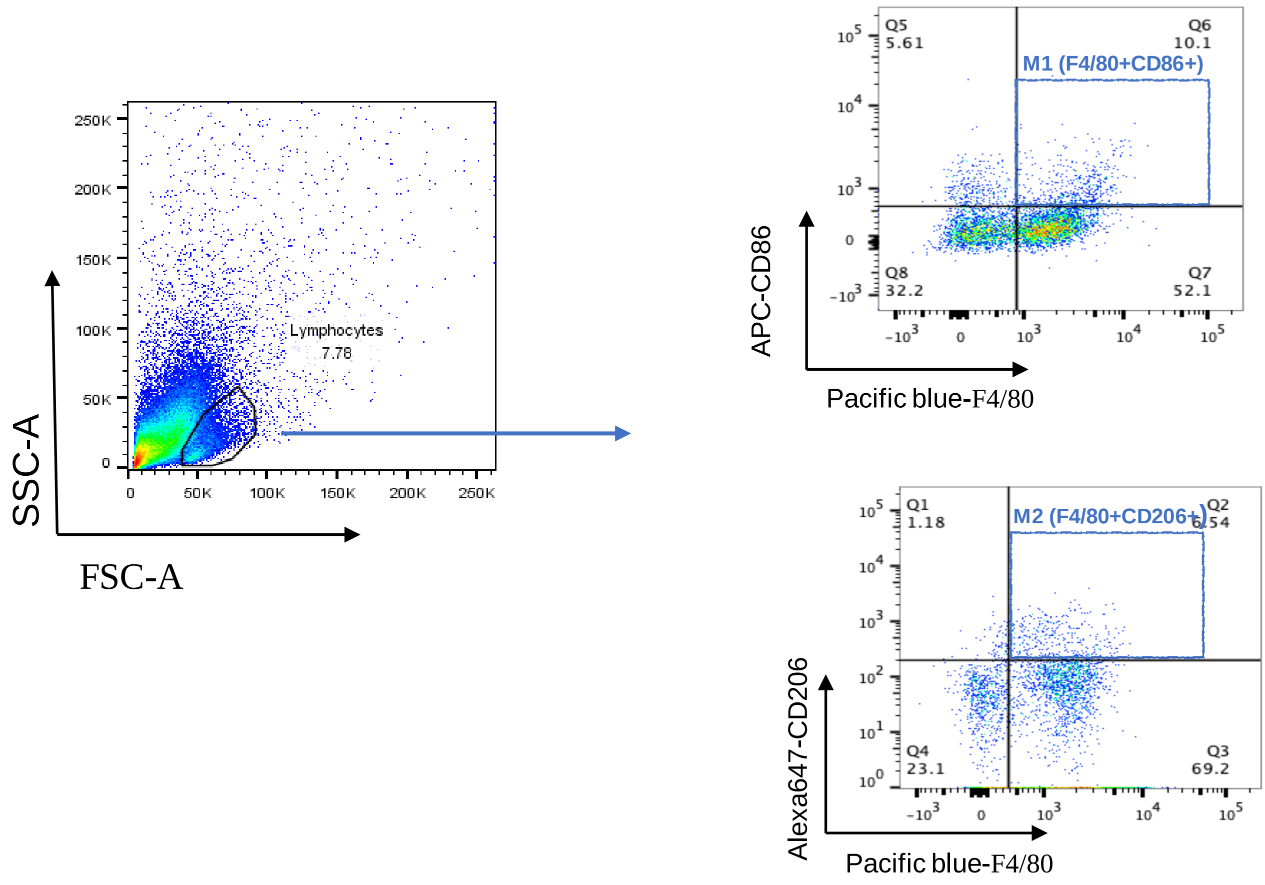

Supplementary Figure 1. Gating strategy for flow cytometry analysis（M1 and M2). The total cells were isolated from the kidney with anti-GBM kidney at day 14 and stained with Pacific blue-conjugated F4/80, APC-conjugated CD86 or Alexa647-conjugated CD206 antibodies and subjected for flow cytometry analysis. Kidney-infiltrating leukocytes were selected according to FSC and SSC intensities as gated in blue in the upper left panel, macrophages were further isolated according to the intensity of F4/80-Pacific blue in the upper right panel with BD FACSAria II Flow Cytometer.

Supplementary Figure 2

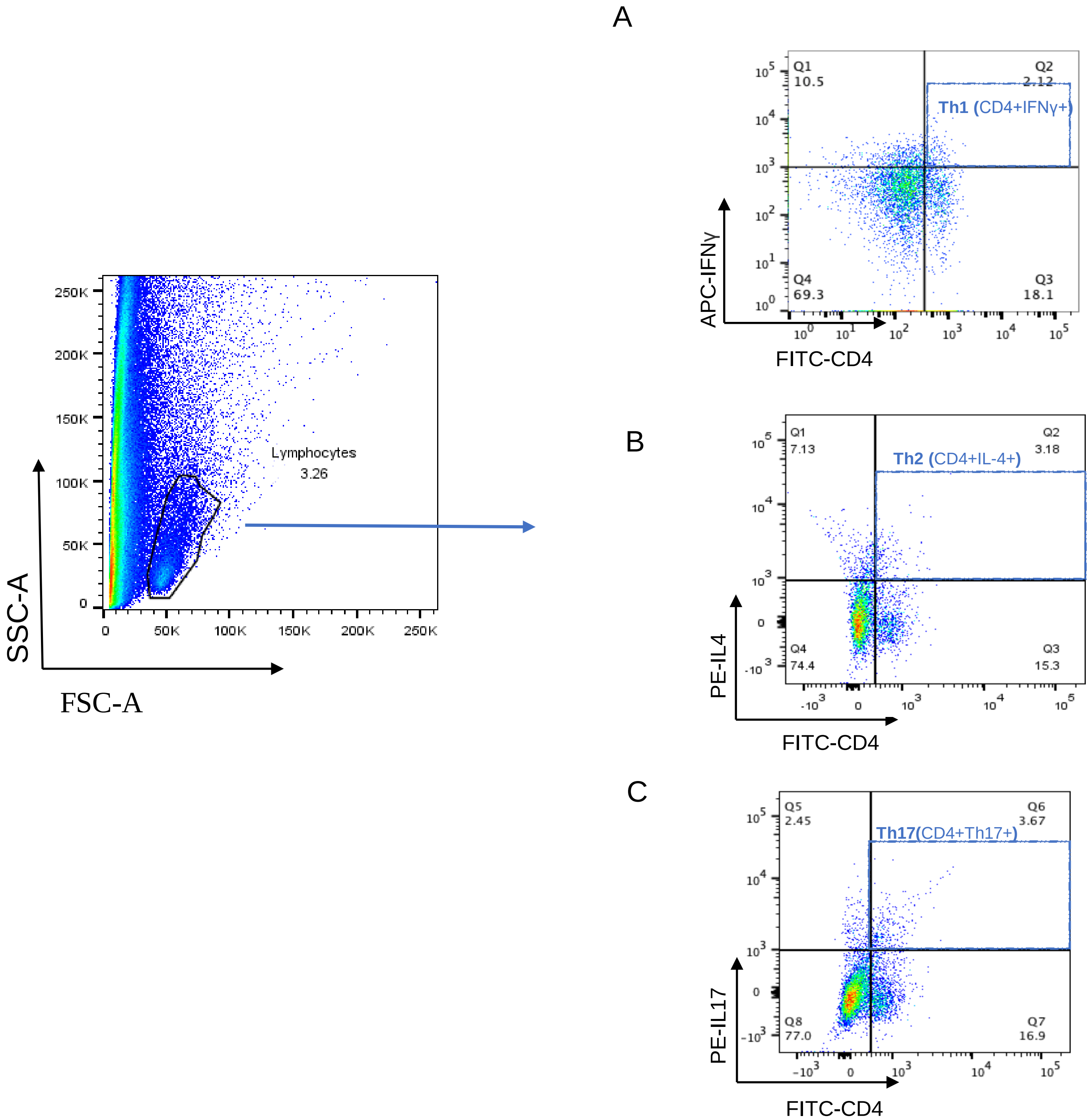

Supplementary Figure 2. Gating strategy for flow cytometry analysis（Th1,Th2and Th17). The total cells were isolated from the kidney with anti-GBM GN at day 14 and stained with FITC-conjugated CD4, APC-conjugated IFNγ or PE-conjugated IL17 antibodies, FITC-conjugated CD4, PE-conjugated IL14antibodies and analyzed by flow cytometry. Kidney-infiltrating leukocytes were selected according to FSC and SSC intensities as gated in blue in the upper left panel. CD4+IFNγ+Th1 cells , CD4+ IL-4+Th2 cells, CD4+ IL-17+Th17 cells were further isolated in the upper right panel with BD FACSAria II Flow Cytometer.

Supplementary Figure 3

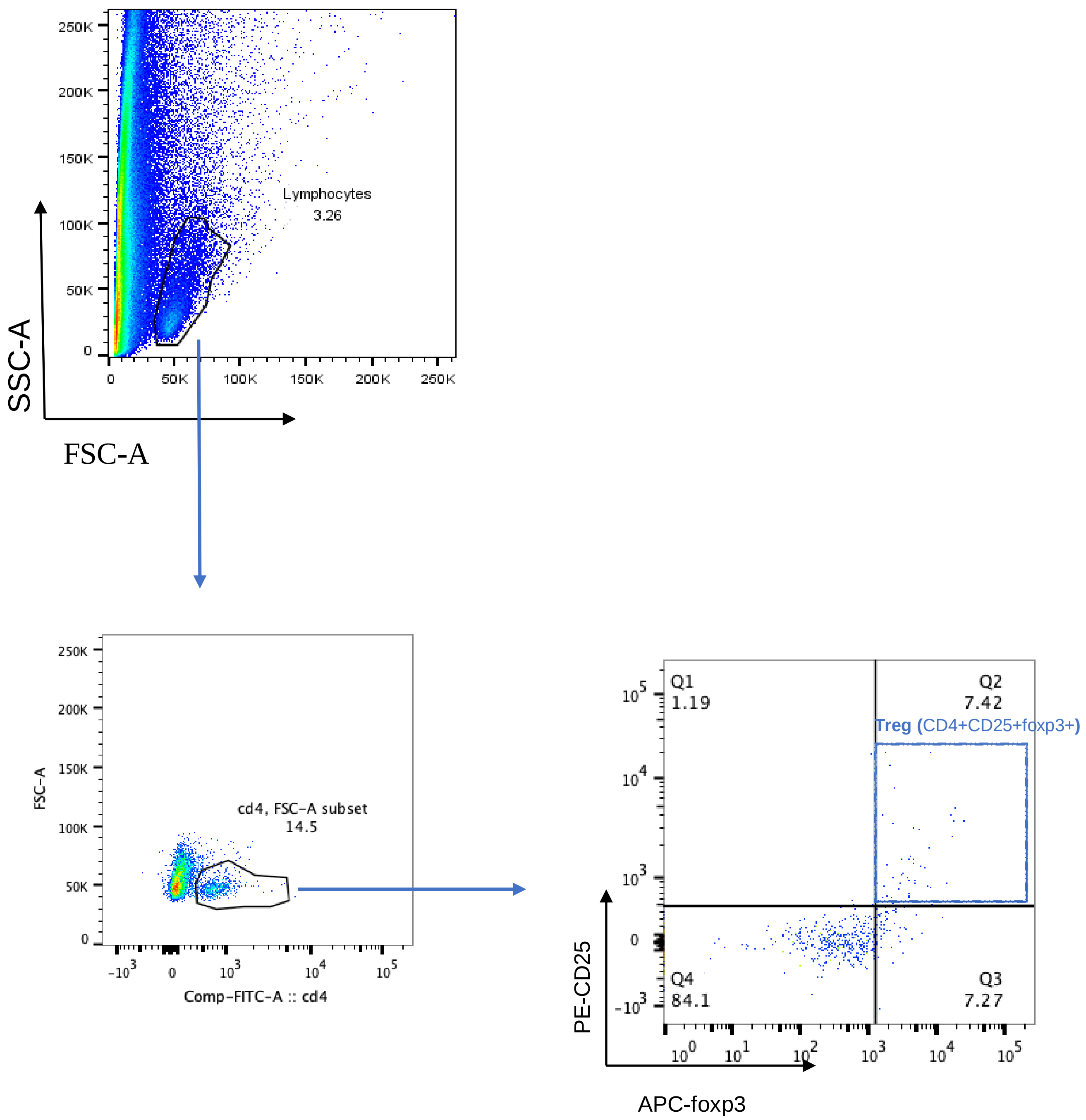

Supplementary Figure 3. Gating strategy for flow cytometry analysis（Treg**).** The total cells were isolated from the kidney with anti-GBM GN at day 14 and stained with FITC-conjugated CD4, APC-conjugated foxp3 and PE-conjugated CD25 antibodies and subjected for flow cytometry analysis. Kidney-infiltrating leukocytes were selected according to FSC and SSC intensities as gated in blue in the upper left panel. Then cells were Gated on CD4+ T cells. CD4+CD25+ Foxp3+Treg were further isolated in the upper right panel with BD FACSAria II Flow Cytometer.
